# The Effect of a Probiotic on Gut Microbiota Stability and Systemic Well-Being during Short-Term Travel

**DOI:** 10.4014/jmb.2510.10037

**Published:** 2026-02-12

**Authors:** Kefeng Yang, Mingxu Yang, Qinghua Yu, Min-Tze Liong, Dongbo Chen, Meiqin Cai

**Affiliations:** 1 Shanghai Jiao Tong University School of Medicine, Shanghai, P.R. China; 2 Xin Hua Hospital Affiliated to Shanghai Jiao Tong University School of Medicine, Shanghai, P.R. China; 3 Laboratory of Microbiology, Immunology, and Metabolism, DiPROBIO (Shanghai) Co., Ltd., Shanghai, P.R. China

**Keywords:** Probiotic, *Bifidobacterium*, Travel, Gut microbiota, Randomized controlled trial

## Abstract

Short-term travel, particularly to new environments, can disrupt gut microbiota homeostasis and induce a range of physical and psychological symptoms. While probiotics are proposed to mitigate these effects, evidence from well-controlled trials during domestic travel, especially along unique routes like China's Silk Road, remains limited. This study investigated the efficacy of a multi-strain *Bifidobacterium* probiotic in maintaining gut microbiota stability and alleviating travel-related symptoms. In a randomized, double-blind, placebo-controlled trial, 74 healthy adults traveling to Xinjiang were assigned to receive either a probiotic (*n* = 39; *B. longum* subsp. *infantis* M-63, *B. breve* M-16V, and *B. longum* BB536, 1.5 × 10^9^ CFU/day) or a placebo (*n* = 35) for five days during travel. Gut microbiota was profiled via metagenomic sequencing (pre- and post-travel), and symptoms were recorded daily. Primary outcomes were changes in gut microbiota composition and function (KEGG pathways). Secondary outcomes included respiratory, gastrointestinal, and systemic symptom scores. Data were analyzed on an intention-to-treat basis. While alpha and beta diversity remained stable in both groups, the probiotic group exhibited a distinct post-travel microbiota enriched with beneficial taxa, including *Bifidobacterium breve* and *Intestinibacillus* at the genus level, and *Lacticaseibacillus rhamnosus*, *Lacticaseibacillus paracasei*, and other *Lacticaseibacillus* species. qPCR confirmed significant increases in administered strains *B. longum* subsp. *infantis* (*p* < 0.001) and *B. breve* (*p* < 0.001). KEGG analysis revealed that the probiotic group maintained a metabolically focused profile (*e.g.*, peptidoglycan biosynthesis, histidine metabolism), whereas the placebo group showed increased abundance of microbial pathways associated with host disease-related signaling (*e.g.*, Huntington disease, various cancers) and inflammatory signaling (*e.g.*, PI3K-Akt signaling pathway). Symptomatically, the probiotic group demonstrated a significantly greater reduction than the placebo in irritability (-92% vs. -31%; *p* = 0.033) and fatigue (-24% vs. +43%; *p* = 0.024) post-travel, and reported less dizziness (-100% vs. -35%; *p* = 0.024). Supplementation with a multi-strain *Bifidobacterium* probiotic during short-term travel promoted the colonization of beneficial bacteria, stabilized gut microbial function against travel-induced dysregulation, and may contribute to supporting systemic well-being during travel.

## Introduction

Global travel represents a significant modern phenomenon, with over 1.3 billion international tourist arrivals recorded in 2023 alone. While travel offers numerous benefits, it simultaneously exposes individuals to a complex array of environmental, dietary, and psychological stressors that can profoundly impact human health [1]. The gastrointestinal tract, housing trillions of microorganisms collectively known as the gut microbiota, appears particularly vulnerable to these travel-associated disruptions [2, 3]. This phenomenon has been increasingly recognized as a significant contributor to various health complaints experienced during and after journeys [4].

The mechanisms underlying travel-induced gut microbiota alterations are multifactorial [5, 6]. Rapid changes in dietary patterns, exposure to novel pathogens, alterations in circadian rhythms, psychological stress, and variations in environmental conditions collectively contribute to microbial community instability [7]. Recent evidence suggests is the emerging understanding that these microbial perturbations extend beyond gastrointestinal symptoms to influence systemic health through the gut-brain axis and immune system modulation [8]. The Silk Road region of Xinjiang, China, presents a unique model for studying these effects, characterized by distinct ethnic diets, significant altitude variations, and dramatic climatic contrasts that collectively pose substantial challenges to gut microbiota homeostasis.

While the association between international travel and traveler's diarrhea has been well-established [3], the impact of domestic travel on gut microbiota composition and function remains inadequately investigated. Furthermore, the spectrum of travel-related health complaints extends beyond gastrointestinal symptoms to include fatigue, irritability, headaches, and other systemic manifestations that significantly impact travel experience and productivity [9]. Current approaches to managing travel-related health issues predominantly focus on symptomatic treatment rather than preventive strategies targeting the underlying microbial changes [4].

Probiotics represent a promising intervention for maintaining gut microbiota homeostasis during travel [10-12]. Specific strains of *Bifidobacterium* have demonstrated particular efficacy in supporting gut barrier function, modulating immune responses, and producing beneficial metabolites [13-15]. However, most existing studies have utilized 16S rRNA sequencing, which provides limited resolution for tracking specific probiotic strains and elucidating functional changes in the microbiome [16, 17]. The current evidence gap necessitates comprehensive investigations using metagenomic sequencing to provide strain-level resolution and functional insights.

This randomized controlled trial was designed to address these critical knowledge gaps by investigating the effects of a multi-strain *Bifidobacterium* probiotic on gut microbiota stability and travel-related symptoms in healthy adults during short-term domestic travel along the Silk Road. We employed metagenomic sequencing and quantitative PCR to achieve three primary objectives: first, to characterize travel-induced changes in gut microbiota composition at high taxonomic resolution; second, to assess functional alterations in microbial community metabolism; and third, to evaluate the efficacy of probiotic supplementation in alleviating both gastrointestinal and systemic travel-related symptoms. We hypothesized that targeted probiotic intervention would enhance microbial resilience, maintain metabolic homeostasis, and consequently reduce the burden of travel-associated health complaints.

## Methods

### Study Design and Ethics Approval

This randomized, double-blind, placebo-controlled clinical trial was conducted according to the Declaration of Helsinki and CONSORT 2010 guidelines. Participants were randomly assigned in a 1:1 ratio to probiotic or placebo groups using a computer-generated sequence by an independent statistician. Both participants and investigators were blinded to group allocation until completion of analyses. The study protocol was approved by the Shanghai Jiao Tong University School of Medicine Public Health and Nursing Research Ethics Committee (Approval No. SJUPN-2025-013-HY1-KS2), and registered at ClinicalTrials.gov (Registration No. NCT07182500, prospective). Written informed consent was obtained from all participants prior to enrolment.

### Participants and Sample Size

Healthy adults aged 18–65 years planning a standardized seven-day round trip to Xinjiang, China were recruited. Exclusion criteria included recent use of antibiotics, probiotics, hormones, or immunosuppressants; chronic systemic disease; major surgery within the previous month; or known allergy to probiotic components. Sample size was calculated based on expected differences in gut microbiota diversity (Cohen’s d = 1.0), requiring 30 participants per arm for 95% power at α = 0.05. Allowing for 20% attrition, 38 participants are needed per group.

### Intervention

Participants received either a probiotic or placebo formulation identical in appearance and packaging. The probiotic contained *B. longum* subsp. *infantis* M-63, *B. breve* M-16V, and *B. longum* BB536 (1.5 × 10^9^ CFU/day) in an oil-based matrix. The placebo contained the same excipients without live bacteria. Participants consumed six drops daily from Day 1 to Day 5 of travel. Compliance was verified by diary logs and returned packaging.

### Samples Collection

Participants recorded daily gastrointestinal and respiratory symptoms and completed standardized questionnaires before departure, during travel, and after return. Two stool samples (~5 g each) were collected from each participant: pre-travel (Day 0) and post-travel (Day 6). Samples were collected using a sterile stool collection kit containing a nucleic acid–stabilizing buffer to preserve microbial composition during transport. Following collection, samples were kept at 4°C, transported to the laboratory within three days, and immediately stored at −80°C until analysis.

### DNA Extraction and Metagenomic Sequencing

DNA was extracted using the NucleoSpin 96 Soil Kit (Macherey-Nagel, France) with bead-beating for cell lysis. DNA purity was verified by Qubit 2.0 fluorometry. Libraries were prepared using the NEBNext Ultra II DNA Library Prep Kit and sequenced (2 × 150 bp) on an Illumina NovaSeq 6000 platform. Reads were trimmed (Trimmomatic v0.36), human sequences filtered (Bowtie2 v2.5), assemblies generated (MEGAHIT v1.2), and genes predicted (Prodigal v2.6). Taxonomic classification used Kraken2 (RefSeq database), and functional annotation was performed via HUMAnN3 with KEGG mapping.

### LEfSe and KEGG Pathway Analysis

Differential taxa were identified using LEfSe analysis (Kruskal–Wallis test; LDA score ≥ 2.0, FDR-adjusted *p* < 0.05). Functional profiling was performed using HUMAnN3 with KEGG mapping, and KEGG pathway abundances were further analyzed in STAMP v2.1 using Welch's t-test.

### Quantitative PCR

Species-specific qPCR assays quantified *Bifidobacterium* taxa using multiplex primers on an ABI 7500 system. Data were expressed as μg/g feces. Amplification efficiencies for all primer–probe sets ranged from 90–105%, with correlation coefficients (R^2^) ≥ 0.99 across standard curves generated from serial dilutions of plasmids containing cloned target sequences. Primer sequences and assay designs were based on previously validated species-specific qPCR methods for *Bifidobacterium* spp. [18], with minor modifications where necessary to optimize multiplex performance. The primer sets used were:


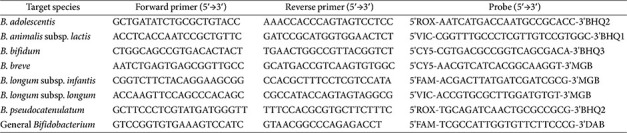


Standard curves were constructed using serial dilutions of plasmids containing cloned target sequences, and results were expressed as μg/g of wet feces.

### Statistical Analysis

All statistical analyses were performed using R (v4.3.2). Alpha diversity indices were calculated using the vegan package, and differences between groups were assessed using the Mann–Whitney U test. Beta diversity was evaluated based on Bray–Curtis dissimilarity and tested using Permutational Multivariate Analysis of Variance (PERMANOVA) with 999 permutations.

Integration, preprocessing, and harmonization of metagenomic sequencing data for taxonomic profiling, KEGG pathway analysis, and LEfSe were performed using Gi-MAPS v2.0 prior to downstream statistical analyses. Differential abundance analyses of taxa and functional pathways were conducted using the statistical methods implemented in LEfSe, with linear discriminant analysis (LDA) used to estimate effect sizes.

Continuous variables were compared between groups using the Mann–Whitney U test. Categorical variables were analyzed using the χ^2^ test or McNemar’s test, as appropriate. Correlations between variables, where applicable, were assessed using Spearman’s rank correlation.

All *p*-values were two-sided, and a *p*-value < 0.05 was considered statistically significant. For analyses involving multiple comparisons, *p*-values were adjusted using the Benjamini–Hochberg false discovery rate (FDR) correction.

## Results

### Participant Recruitment, Baseline Characteristics, and Intervention Compliance

**Fig. 1:**
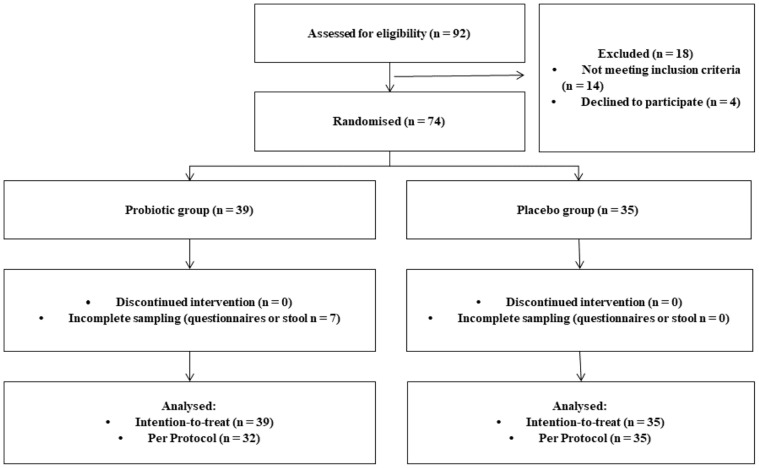
CONSORT flow of participant screening, randomization, allocation, and analysis inclusion. Of 92 screened individuals, 74 were randomized and included in final analysis (39 probiotic, 35 placebo). No withdrawals or adverse events occurred, confirming good compliance. The study maintained high adherence and data completeness across participants. CONSORT = Consolidated Standards of Reporting Trials.

The clinical trial successfully enrolled 74 participants through a comprehensive screening process of 92 potentially eligible volunteers. The participant flow diagram (Fig. 1) illustrates the complete recruitment and randomization pathway, with 39 participants allocated to the probiotic intervention group and 35 to the placebo control group. All randomized participants were included in the final intention-to-treat (ITT) analysis, ensuring the preservation of randomization benefits and enhancing the generalizability of our findings.

In cases where participants had incomplete symptom diaries or missing stool samples, all available data were retained for the ITT analysis without imputation; participants remained in their originally assigned groups, and outcomes were analyzed using an observed-case approach. Overall, symptom diary data were complete for 67 of 74 participants (90.5%). Missing symptom diary data occurred in 7 participants (9.5%), all of whom were in the probiotic group (7/39, 17.9%), while no missing symptom diary data were observed in the placebo group (0/35, 0%). No stool samples were missing in either group, resulting in 100% completeness for microbiome analyses.

The distribution of missing symptom diary data was therefore unbalanced between groups and was explicitly accounted for by using an ITT observed-case approach without imputation. As missingness was confined to symptom diary outcomes and no biological samples were missing, the integrity of microbiome-related analyses was not affected.

Baseline demographic and clinical characteristics demonstrated excellent comparability between the intervention groups (Table 1), as assessed using the Mann–Whitney U test for continuous variables and the χ^2^ test for categorical variables. The study population comprised adults with mean age: probiotic group 39.22 ± 1.18 years, placebo group 36.21 ± 0.91 years (Mann–Whitney U test, *p* = 0.070), with balanced sex distribution (probiotic: 43.6% male, placebo: 31.4% male; χ^2^ test, *p* = 0.403) and comparable body mass indices (probiotic: 23.35 ± 0.66 kg/m^2^, placebo: 23.33 ± 0.54 kg/m^2^; Mann–Whitney U test, *p* = 0.607). Comprehensive assessment of additional variables including ethnicity, household registration, marital status, education level, household income, smoking status, and self-rated health status revealed no statistically significant differences between groups, confirming the success of the randomization procedure and establishing a robust foundation for subsequent intervention effect analyses.

Intervention compliance was rigorously monitored throughout the study period using multiple verification methods including daily diary logs, returned product packaging quantification, and participant interviews. The results indicated excellent adherence to the study protocol, with no reported deviations in intervention administration timing or dosage. Critically, no adverse events related to the study product were reported, and there was no concomitant use of excluded medications such as antibiotics, additional probiotics, or immunosuppressants during the trial period, ensuring the integrity of the intervention assessment.

### Probiotic Supplementation Significantly Improves Systemic Well-being during Travel

Comprehensive symptom monitoring throughout the travel period revealed distinct patterns of symptom expression between the intervention groups (Table 2). Baseline assessment (Day 0) confirmed comparable symptom profiles across all categories, with no statistically significant differences in respiratory, gastrointestinal, or systemic symptom scores between groups, thereby establishing equivalent baseline health status, as assessed using the Mann–Whitney U test for between-group comparisons of symptom scores.

During the active travel period (Days 1-5), both groups maintained generally low levels of respiratory symptoms, with no significant between-group differences observed for cough (probiotic: 0.07 ± 0.04, placebo: 0.08 ± 0.04; *p* = 0.758), runny nose (probiotic: 0.10 ± 0.05, placebo: 0.12 ± 0.06; *p* = 1.000), or sore throat (probiotic: 0.08 ± 0.04, placebo: 0.09 ± 0.04; *p* = 0.806). Similarly, gastrointestinal symptoms including loose stools (probiotic: 0.21 ± 0.06, placebo: 0.10 ± 0.04; *p* = 0.134), abdominal pain (probiotic: 0.02 ± 0.01, placebo: 0.08 ± 0.04; *p* = 0.397), and constipation (probiotic: 0.16 ± 0.06, placebo: 0.17 ± 0.06; *p* = 0.849) showed comparable incidence between groups during travel, with all between-group comparisons conducted using the Mann–Whitney U test.

The most pronounced and statistically significant differences were observed in the analysis of systemic symptoms. The probiotic group demonstrated a substantially greater reduction in irritability from baseline to post-travel (Day 6), with a mean change of -0.35 ± 0.08 compared to -0.09 ± 0.10 in the placebo group (*p* = 0.033), as assessed using the Mann–Whitney U test. For fatigue symptoms, a particularly striking contrast was observed: the probiotic group showed significant improvement (-0.15 ± 0.10), while the placebo group experienced worsening symptoms (0.23 ± 0.15), resulting in a highly significant between-group difference (*p* = 0.024), based on the Mann–Whitney U test of change scores. At the post-travel assessment, the probiotic group reported complete absence of dizziness (0.00 ± 0.00), significantly lower than the placebo group (0.17 ± 0.08; *p* = 0.024). Additionally, a strong trend toward reduction in headache incidence was observed in the probiotic group at Day 6 (0.03 ± 0.03 vs. 0.17 ± 0.06 in placebo; *p* = 0.054).

Taken altogether, the probiotic and placebo groups were well-matched at baseline. During and after travel, no significant differences were observed between the groups for the majority of respiratory and gastrointestinal symptoms. However, the probiotic intervention was associated with statistically significant improvements in systemic symptoms post-travel.

### Gut Microbiota Composition

**Fig. 2:**
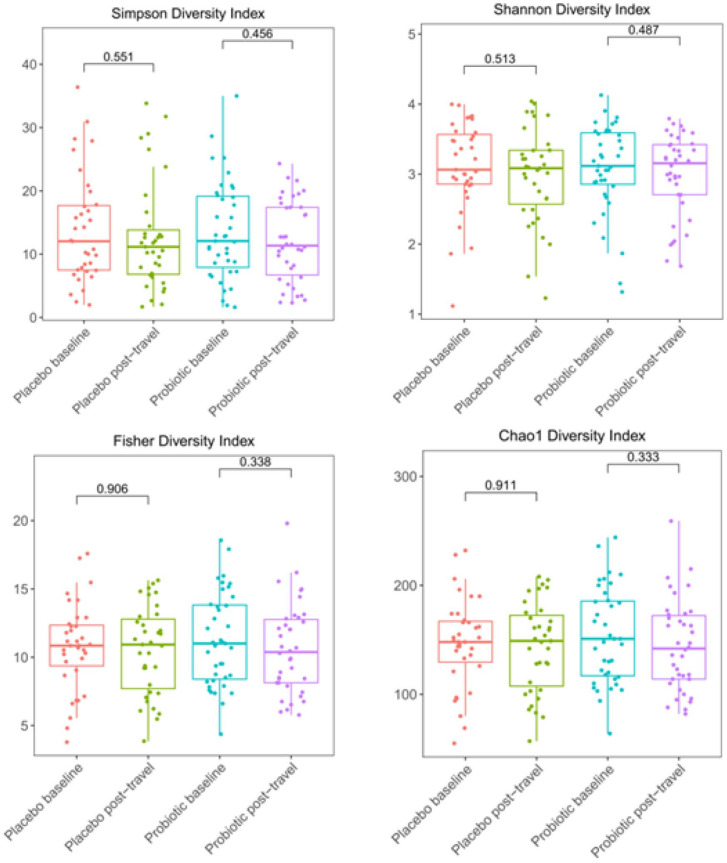
Alpha diversity indices (Simpson, Shannon, Fisher, Chao1) before and after travel. Boxplots depict microbial diversity within each group; line inside the box represents the median; whiskers represent the lowest and highest values within the interquartile range (IQR). Diversity remained stable in both groups. The Mann–Whitney U test was used for group comparisons; *p* < 0.05 significant.

**Alpha and beta diversity.** Analysis of microbial community diversity revealed remarkable stability during the short-term travel period. Alpha diversity assessments using multiple indices including Chao1 (richness), Shannon (diversity), Fisher, and Simpson indices demonstrated no significant changes within or between groups (all *p* > 0.05; Fig. 2), as assessed using the Mann–Whitney U test, indicating preservation of microbial community complexity despite environmental perturbations.

**Fig. 3:**
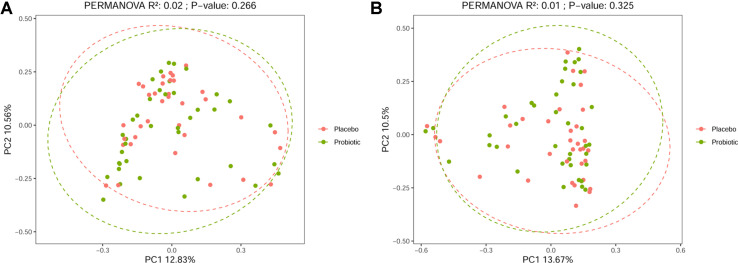
Beta diversity analysis of gut microbial community structure. PCoA plots based on Bray-Curtis distance show overlapping clusters between probiotic and placebo groups (**A**) before and (**B**) after travel. No significant differences detected by PERMANOVA (*p* > 0.05). Overall microbial community structure remained stable in both groups, suggesting resilience to short-term travel stress. PCoA = principal coordinate analysis; PERMANOVA = Permutational Multivariate Analysis of Variance.

Beta diversity analysis based on Bray-Curtis dissimilarity metrics, visualized through principal coordinate analysis (PCoA), revealed overlapping clustering patterns between intervention groups and time points (Fig. 3). Permutational multivariate analysis of variance (PERMANOVA), based on Bray–Curtis distance matrices, confirmed the absence of significant overall compositional divergence between groups (*p* > 0.05), suggesting that the fundamental structure of the microbial communities remained stable throughout the study period.

**Taxonomic profiles.** Despite this overall stability, sophisticated differential abundance analysis using Linear Discriminant Analysis Effect Size (LEfSe), which applies the non-parametric Kruskal–Wallis test followed by linear discriminant analysis (LDA) to estimate effect size, identified specific, statistically significant taxonomic shifts associated with probiotic intervention. The post-travel microbiota of the probiotic group showed increased post-travel abundance of beneficial taxa including *B. breve* and *Intestinibacillus*. At the species level, this enrichment was characterized by significant increases in *Lacticaseibacillus rhamnosus*, *Lacticaseibacillus paracasei*, and other *Lacticaseibacillus* species, confirming successful colonization and dominance of the supplemented probiotic strains.

In contrast, the placebo group's baseline microbiota exhibited higher abundance of genera including *Streptococcus* and *Gemella*. While the placebo group showed post-travel increases in potentially beneficial taxa such as *Faecalibacterium* (species *Faecalibacterium prausnitzii*) and *Lactobacillus* (species *Lactobacillus delbrueckii*), these changes occurred within a context of broader functional dysregulation as revealed by subsequent pathway analysis.[Fig F4][Fig F5]

### Quantitative PCR Validation Confirms Strain-Specific Enhancement

To complement the metagenomic findings and provide precise quantification of *Bifidobacterium* dynamics, we implemented species-specific quantitative PCR assays targeting major *Bifidobacterium* species. While total *Bifidobacterium* abundance at the genus level remained comparable between groups, targeted analysis revealed profound differences at the species level that aligned with the probiotic formulation.

The administered strains showed remarkable post-travel increases in the probiotic group, with *B. longum* subsp. *infantis* demonstrating highly significant enrichment (*p* < 0.001) and B. breve showing equally substantial enhancement (*p* < 0.001) compared to the placebo group, as assessed using the Mann–Whitney U test. We also observed a significant increase in *B. animalis* subsp. *lactis* (*p* = 0.006) in the probiotic group, despite this species not being included in the intervention. While the underlying mechanism cannot be determined from the current data, this pattern may reflect indirect ecological effects within the gut environment. However, such explanations remain hypothetical and require further investigation.

### Probiotic Maintenance of Metabolic Homeostasis and Prevention of Functional Dysregulation

**Fig. 6:**
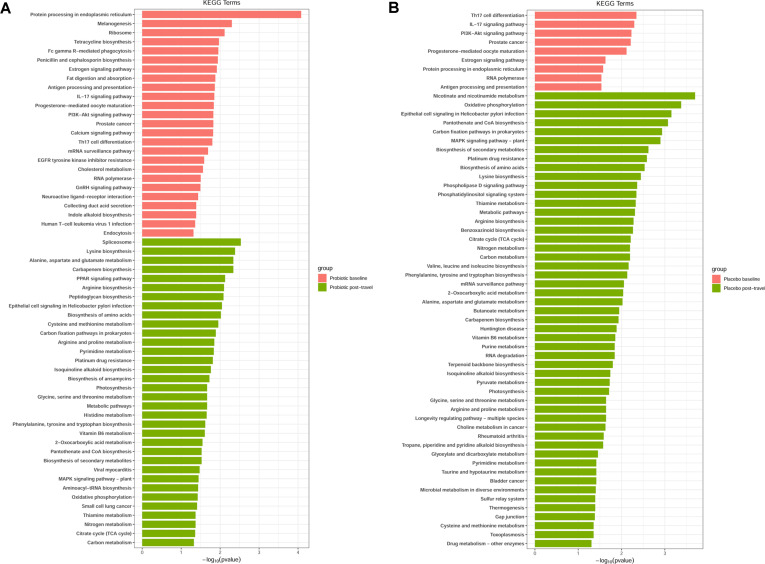
Differential KEGG pathway enrichment in the gut microbiome of participants receiving (A) probiotics or (B) placebo. KEGG pathway analysis revealed that probiotic supplementation maintained a stable, metabolically focused gut microbiome post-travel, whereas the placebo group showed increased abundance of microbial pathways associated with host disease-related signaling, inflammatory signaling, and metabolic stress. Top 100 enriched pathways (*p* < 0.05) per group are shown. Statistical analysis was performed using the Welch’s t-test within STAMP software (*p* < 0.05). KEGG = Kyoto Encyclopedia of Genes and Genomes.

Comprehensive functional annotation using KEGG pathway analysis revealed clear differences in microbial community functional potential between intervention groups (Fig. 6), as assessed using Welch's t-test in STAMP for pathway relative abundances. The probiotic group's gut microbiome maintained a more conserved metabolic profile, characterized by higher representation of core biosynthetic pathways, including peptidoglycan biosynthesis, biosynthesis of ansamycins, and histidine metabolism, suggesting greater stability of fundamental microbial functions during travel.

In contrast, the placebo group showed broader shifts across multiple KEGG pathway categories following travel. Notably, several pathways annotated under the KEGG “Human Diseases” classification exhibited increased relative abundance. Because these annotations often represent conserved microbial gene modules rather than indicators of clinical disease risk, these findings should be interpreted as reflecting generalized stress- or perturbation-associated changes in functional gene content rather than activation of disease-specific processes. Similarly, the observed enrichment of pathways related to Environmental Information Processing, including phospholipase D signaling, calcium signaling, Notch signaling, and PI3K-Akt signaling, likely represent shifts in microbial community signaling and adaptation rather than host-derived pathological pathways.

Overall, although KEGG disease-associated annotations do not imply direct clinical disease mechanisms, the divergence between groups suggests that the probiotic intervention helped maintain a more functionally stable gut microbiome under travel-related environmental stress. This functional stability aligns with, but does not mechanistically dictate, the reduced systemic symptoms observed in the probiotic group.

## Discussion

### Comprehensive Summary of Key Findings

This randomized controlled trial provides evidence that supplementation with a multi-strain *Bifidobacterium* probiotic confers multi-level benefits during short-term travel to novel environments. Our findings demonstrate a clear hierarchy of effects, beginning with successful microbial colonization and extending to functional stabilization and ultimately clinical symptom improvement. The probiotic intervention resulted in the increased abundances of administered strains post-travel (*B. longum* subsp. *infantis* and *B. breve*) while simultaneously promoting the enrichment of beneficial *Lacticaseibacillus* species, which may reflect ecological interactions within the microbiome.

Most notably, our functional metagenomics analysis revealed that the probiotic attenuated the increase in pathways linked to disease-related signaling that were prominently observed in the placebo group. This protective effect at the functional level represents a crucial advancement in understanding how probiotics exert their benefits beyond simple taxonomic changes. The clinical translation of this microbial stabilization was clearly demonstrated through significant improvements in systemic symptoms, with the probiotic group exhibiting substantially greater reductions in fatigue and irritability, along with complete resolution of dizziness symptoms post-travel.

### Integration with Current Scientific Literature

Our findings regarding the alleviation of non-gastrointestinal symptoms through probiotic intervention substantially strengthen the evolving evidence base supporting the gut-brain axis [19]. While previous travel-focused probiotic research has predominantly emphasized diarrheal outcomes [6, 11], our results reveal that the benefits extend considerably beyond gastrointestinal symptoms to encompass critical quality-of-life indicators. The pronounced reduction in fatigue observed in our probiotic group, particularly when contrasted with the worsening fatigue in the placebo group, resonates strongly with emerging research in stress physiology and chronic fatigue management [20]. This parallel suggests that travel-induced stress and other forms of physiological exhaustion may share common pathways that are amenable to probiotic intervention.

The functional metagenomics data generated in this study provide unprecedented mechanistic insights into travel-related microbial perturbations. The observation that travel stress in the placebo group activated pathways associated with cancer progression, neurodegenerative conditions, and inflammatory signaling represents a novel contribution to travel medicine literature [21]. This pattern suggests that changes in gut microbiota constitutes not merely a compositional rearrangement but rather a fundamental functional aberration wherein the microbial community's metabolic activities shift toward potentially detrimental processes [5, 9]. The probiotic's capacity to suppress this widespread functional dysregulation provides compelling support for the concept of microbiome-resilience and demonstrates how targeted microbial interventions can maintain ecological stability under environmental challenge [22, 23].

### Elaborated Mechanistic Framework

The beneficial effects observed in this study likely operate through an interconnected multi-mechanistic framework. The enrichment of *Bifidobacterium* and *Lacticaseibacillus* species, both established producers of short-chain fatty acids (SCFAs) including acetate, lactate, and possibly butyrate, probably enhanced gut barrier integrity through tight junction protein modulation and goblet cell stimulation [24, 25]. These SCFAs simultaneously function as potent immunomodulators, potentially regulating host immune responses and metabolic signaling pathways that influence systemic inflammation [26].

The unanticipated increase in *B. animalis* subsp. *lactis* abundance within the probiotic group, despite its absence from the administered formulation, strongly suggests sophisticated cross-feeding interactions. This phenomenon likely involves metabolic cooperation where fermentation end-products (particularly lactate) from the supplemented strains provided substrates for secondary fermenting bacteria, thereby creating a synergistic ecological network that enhanced overall microbial community stability [27].

The prevention of inflammatory pathway activation (specifically PI3K-Akt and IL-17 signaling) in the probiotic group probably contributed substantially to the observed systemic symptom improvements [28]. Chronic low-grade inflammation represents a well-established driver of fatigue and mood disturbances, potentially through cytokine-mediated effects on neurotransmitter metabolism, neuroendocrine function, and mitochondrial efficiency [29]. By maintaining a metabolically focused, non-inflammatory gut microbiome profile, the probiotic intervention may be associated with reduced activation of inflammatory signaling pathways thereby protecting against travel-induced malaise, irritability, and dizziness [30, 31].

Additional mechanisms may involve microbial influence on tryptophan metabolism, with potential effects on serotonin and kynurenine pathway regulation, and modulation of the hypothalamic-pituitary-adrenal (HPA) axis responsiveness [32, 33]. The specific strain combination utilized in this study may have also enhanced mucin production and reinforced the gut barrier, thereby reducing bacterial translocation and subsequent immune activation [34].

### Comprehensive Strengths and Limitations Assessment

This study exhibits several notable methodological strengths that enhance the validity and impact of its findings. The randomized, double-blind, placebo-controlled design effectively minimizes selection bias and confounding. The utilization of high-resolution metagenomic sequencing enabled comprehensive taxonomic and functional profiling beyond the capabilities of 16S rRNA sequencing, while species-specific qPCR provided precise quantification and verification of strain-level colonization. The intention-to-treat analysis approach conserved randomization benefits and enhanced the real-world applicability of our results. Several limitations warrant consideration when interpreting these findings. The relatively short travel duration and observation period leave the long-term persistence of the probiotic effects uncertain. The exclusive focus on healthy adults limits generalizability to pediatric, elderly, or immunocompromised populations. While we identified functional pathway alterations, direct quantification of fecal SCFA concentrations, inflammatory biomarkers, and gut permeability measures would have strengthened the mechanistic connections between microbial changes and clinical outcomes. Furthermore, the study was conducted in a specific geographic and cultural context (Silk Road travel), which may limit direct extrapolation to other travel environments.

In addition, the subjective nature of symptom reporting, despite standardized instruments, remains vulnerable to recall and reporting biases. Several of the systemic symptoms that showed group differences, such as fatigue, irritability, and dizziness, are inherently subjective and can be influenced by numerous external factors, including sleep quality, daily schedule, stress, hydration status, and environmental conditions such as altitude or climate. Although participants were instructed to report symptoms consistently, we cannot fully disentangle the effects of these external variables from the impact of the probiotic intervention. As a result, the observed reductions in subjective symptoms should be interpreted with caution, and future studies incorporating objective physiological markers or controlled travel conditions would help clarify the extent to which probiotics directly modulate these outcomes.

Future research directions should include longer follow-up periods to assess durability of effects, incorporation of objective biomarker measurements, investigation in diverse population groups, and exploration of dose-response relationships. Studies examining the interaction between specific probiotic strains and dietary components during travel would further elucidate the mechanisms underlying the observed benefits.

This comprehensive investigation establishes that targeted probiotic supplementation represents an effective strategy for maintaining gut microbiome homeostasis during travel, with benefits extending beyond gastrointestinal symptom prevention to encompass functional stabilization and meaningful improvements in overall well-being.

## Conclusion

In conclusion, these findings suggest that probiotic intervention may support microbiota stability. It goes beyond preventing diarrhea by demonstrating a protective effect against gut microecology and significant improvements in systemic well-being. These findings position specific probiotic strains as a valuable non-pharmacological tool for promoting resilience in the face of travel-related stressors.

## Figures and Tables

**Fig. 4 F4:**
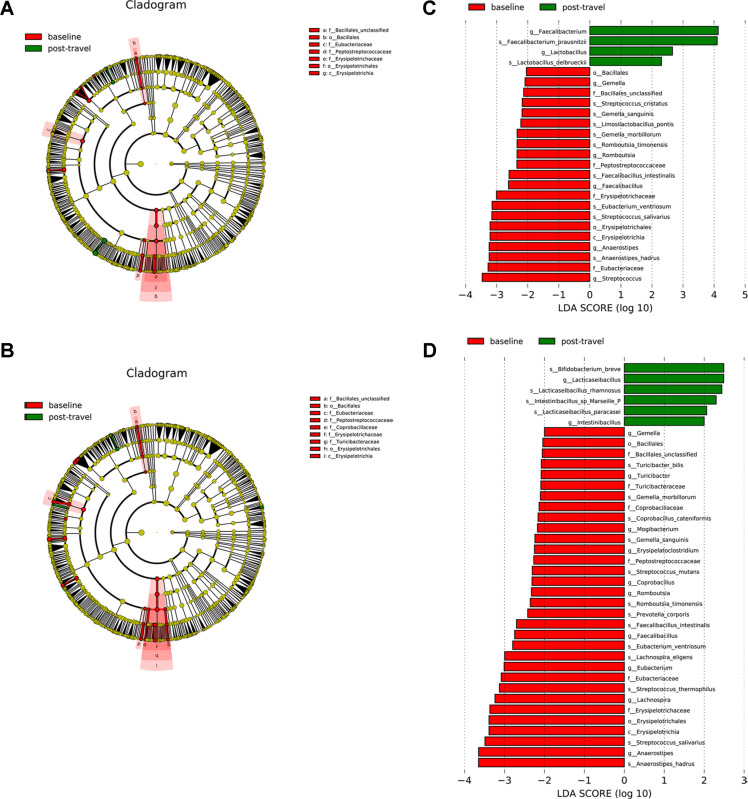
LEfSe-based identification of differentially abundant taxa before and after travel. Cladogram and LDA bar plots highlight taxa enriched in the placebo (A,C) and probiotics (B,D) groups. *Bifidobacterium* increased in the probiotic group, while *Faecalibacterium* expanded in placebo. LDA threshold > 2.0, *p* < 0.05. LDA = linear discriminant analysis; LEfSe = Linear Discriminant Analysis Effect Size.

**Fig. 5 F5:**
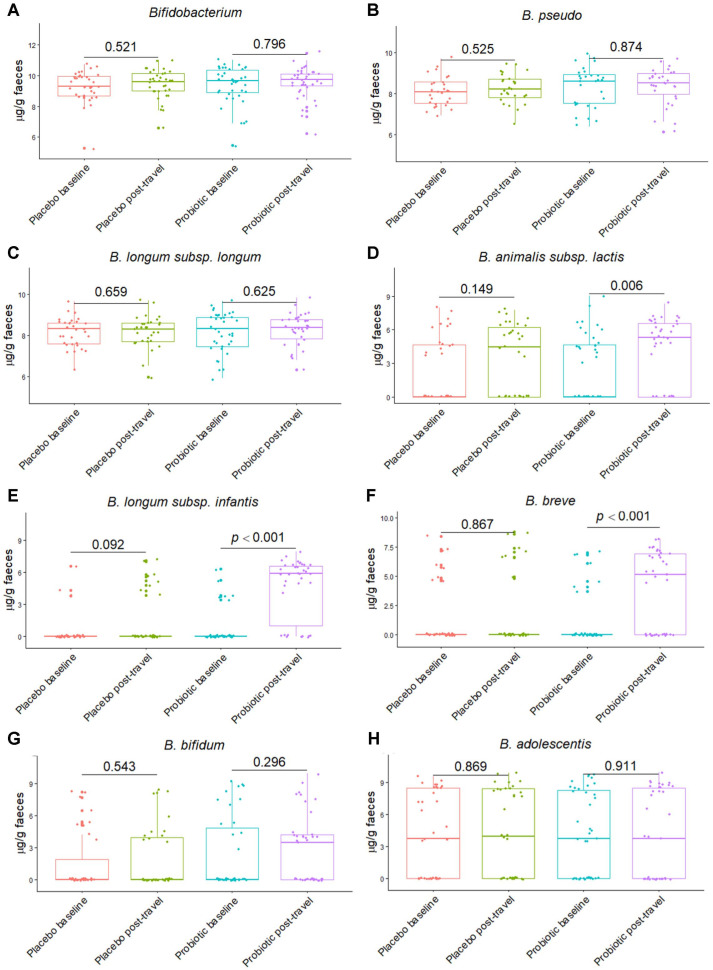
Quantitative profiling of fecal *Bifidobacterium* species before and after short-term travel. Overall abundance of total *Bifidobacterium* remained stable in both groups. Values are expressed as μg/g feces determined by qPCR for: (**A**) total *Bifidobacterium*, (**B**) *B. pseudocatenulatum* (**C**) *B. longum* subsp. longum, (**D**) *B. animalis* subsp. *lactis*, (**E**) *B. longum* subsp. *infantis*, (**F**) *B. breve*, (**G**) *B. bifidum*, and (**H**) *B. adolescentis*. Boxes denote interquartile range with median lines; whiskers indicate minima and maxima. Comparisons were performed using the Mann–Whitney U test.

**Table 1:** Baseline demographic and clinical characteristics of participants in the probiotic and placebo groups.

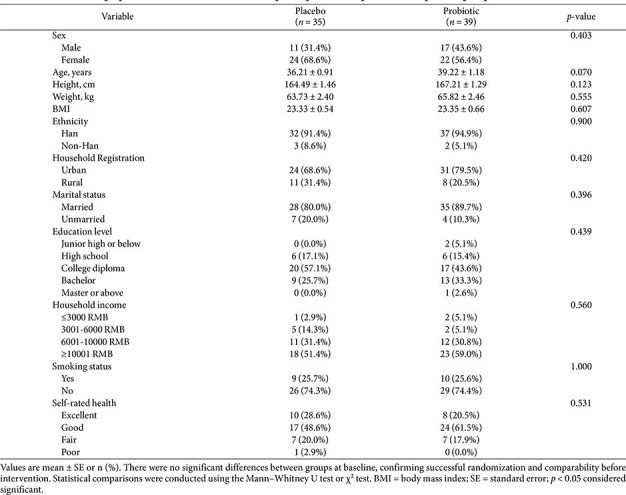

**Table 2:** Mean symptom scores for respiratory, gastrointestinal, and systemic symptoms during short-term travel.

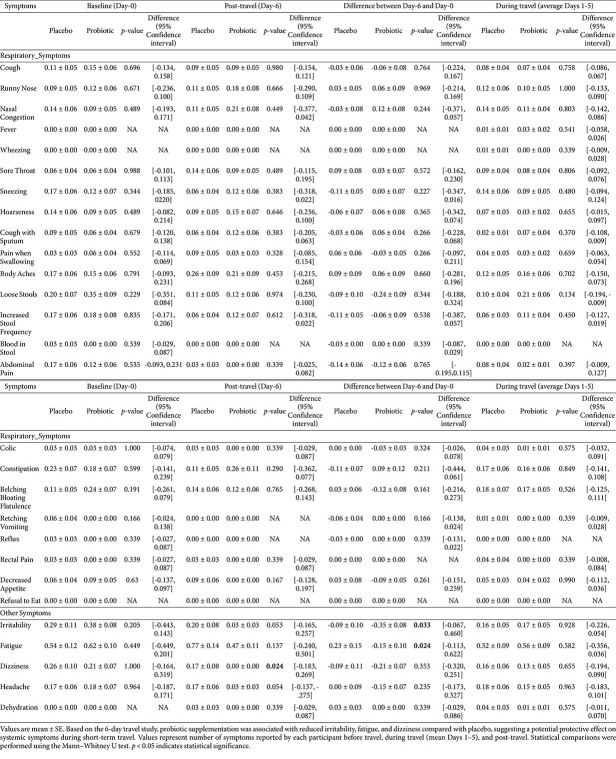

## References

[1] Henares D, Monsalvez V, Brotons P, Machado ML, Capilla S, Gomila-Grange A (2024). Human gut microbiota composition associated with international travels. Travel Med. Infect. Dis..

[2] Zhao Y, Li C, Wu K, Chen H, Wang Q, Xiao Y (2024). Exploring the impact of short term travel on gut microbiota and probiotic bacteria mediated stability. Biomedicines.

[3] Boolchandani M, Blake KS, Tilley DH, Cabada MM, Schwartz DJ, Patel S (2022). Impact of international travel and diarrhea on gut microbiome and resistome dynamics. Nat. Commun.

[4] Butler D, McLoughlin R, Flaherty GT (2022). Travel‐related gastrointestinal diseases: assessment and management. Public Health Chall..

[5] Worby CJ, Sridhar S, Turbett SE, Becker MV, Kogut L, Sanchez V (2023). Gut microbiome perturbation, antibiotic resistance, and *Escherichia coli* strain dynamics associated with international travel: a metagenomic analysis. Lancet Microbe.

[6] Youmans BP, Ajami NJ, Jiang Z-D, Campbell F, Wadsworth WD, Petrosino JF (2015). Characterization of the human gut microbiome during travelers' diarrhea. Gut Microbes.

[7] Bourdeau-Julien I, Castonguay-Paradis S, Rochefort G, Perron J, Lamarche B, Flamand N (2023). The diet rapidly and differentially affects the gut microbiota and host lipid mediators in a healthy population. Microbiome.

[8] Yang J, Kim H-D, Barrila J, Lee S-H, Nickerson CA, Ott CM (2025). Navigating mental health in space: gut-brain axis and microbiome dynamics. Exp. Mol. Med..

[9] Abdullah ASM, Hamer DH (2006). Travel-related health problems of Hong Kong residents: assessing the need for travel medicine services. Travel Med. Infect. Dis.

[10] Srivastava AK, Rohil V, Bhushan B, Eslavath MR, Gupta H, Chanda S (2021). Probiotics maintain the gut microbiome homeostasis during Indian Antarctic expedition by ship. Sci. Rep..

[11] Alharbi BF, Alateek AA (2024). Investigating the influence of probiotics in preventing Traveler's diarrhea: meta-analysis based systematic review. Travel Med. Infect. Dis..

[12] McFarland LV, Goh S (2019). Are probiotics and prebiotics effective in the prevention of travellers' diarrhea: a systematic review and meta-analysis. Travel Med. Infect. Dis..

[13] Abdulqadir R, Engers J, Al-Sadi R (2023). Role of *Bifidobacterium* in modulating the intestinal epithelial tight junction barrier: current knowledge and perspectives. Curr. Dev. Nutr.

[14] Gavzy SJ, Kensiski A, Lee ZL, Mongodin EF, Ma B, Bromberg JS (2023). *Bifidobacterium* mechanisms of immune modulation and tolerance. Gut Microbes.

[15] Kim G, Yoon Y, Park JH, Park JW, Noh M-G, Kim H (2022). Bifidobacterial carbohydrate/nucleoside metabolism enhances oxidative phosphorylation in white adipose tissue to protect against diet-induced obesity. Microbiome.

[16] Kampmann C, Dicksved J, Engstrand L, Rautelin H (2021). Changes to human faecal microbiota after international travel. Travel Med. Infect. Dis..

[17] Gandasegui J, Vergara A, Fleitas P, Rubio E, Fernandez-Pittol M, Aylagas C (2024). Gut microbiota composition in travellers is associated with faecal lipocalin-2, a mediator of gut inflammation. Front. Cell. Infect. Microbiol..

[18] Kim H-B, Kim E, Yang S-M, Lee S, Kim M-J, Kim H-Y (2020). Development of Real-Time PCR Assay to Specifically Detect 22 *Bifidobacterium* Species and Subspecies Using Comparative Genomics. Front. Microbiol..

[19] Johnson KV-A, Steenbergen L (2025). Probiotics reduce negative mood over time: the value of daily self-reports in detecting effects. NPJ Ment. Health Res..

[20] Hsu C-Y, Ahmad I, Maya RW, Abass MA, Gupta J, Singh A (2025). The potential therapeutic approaches targeting gut health in myalgic encephalomyelitis/chronic fatigue syndrome (ME/CFS): a narrative review. J. Transl. Med..

[21] McIntosh IB, Swanson V, Power KG, Raeside F, Dempster C (1998). Anxiety and health problems related to air travel. J. Travel Med..

[22] Chandrasekaran P, Weiskirchen S, Weiskirchen R (2024). Effects of probiotics on gut microbiota: an overview. Int. J. Mol. Sci..

[23] McFarland LV (2007). Meta-analysis of probiotics for the prevention of traveler's diarrhea. Travel Med. Infect. Dis..

[24] Usta-Gorgun B, Yilmaz-Ersan L (2020). Short-chain fatty acids production by *Bifidobacterium* species in the presence of salep. Electron. J. Biotechnol..

[25] Thananimit S, Pahumunto N, Teanpaisan R (2022). Characterization of short chain fatty acids produced by selected potential probiotic *Lactobacillus* strains. Biomolecules.

[26] Mann ER, Lam YK, Uhlig HH (2024). Short-chain fatty acids: linking diet, the microbiome and immunity. Nat. Rev. Immunol..

[27] Vizioli C, Jaime-Lara R, Daniel SG, Franks A, Diallo AF, Bittinger K (2023). Administration of *Bifidobacterium* animalis subsp. *lactis* strain BB-12^®^ in healthy children: characterization, functional composition, and metabolism of the gut microbiome. Front. Microbiol..

[28] Chen L, Deng H, Cui H, Fang J, Zuo Z, Deng J (2018). Inflammatory responses and inflammation-associated diseases in organs. Oncotarget.

[29] Safadi JM, Quinton AMG, Lennox BR, Burnet PWJ, Minichino A (2022). Gut dysbiosis in severe mental illness and chronic fatigue: a novel trans-diagnostic construct? A systematic review and meta-analysis. Mol. Psychiatry.

[30] Merkouris E, Mavroudi T, Miliotas D, Tsiptsios D, Serdari A, Christidi F (2024). Probiotics' effects in the treatment of anxiety and depression: a comprehensive review of 2014-2023 clinical trials. Microorganisms.

[31] Dubois T, Zdanowicz N, Jacques D, Lepiece B, Jassogne C (2023). Microbiota diversity and inflammation as a new target to improve mood: probiotic use in depressive disorder. Psychiatr. Danub..

[32] Agus A, Planchais J, Sokol H (2018). Gut microbiota regulation of tryptophan metabolism in health and disease. Cell Host Microbe.

[33] Shen X, Mu X (2024). Systematic insights into the relationship between the microbiota-gut-brain axis and stroke with the focus on tryptophan metabolism. Metabolites.

[34] Abe F, Muto M, Yaeshima T, Iwatsuki K, Aihara H, Ohashi Y (2010). Safety evaluation of probiotic bifidobacteria by analysis of mucin degradation activity and translocation ability. Anaerobe.

